# LGBITQ+ INCLUSIVITY IN GERIATRIC CARE IN NORWAY: ATTITUDES AND PRACTICES AMONG HEALTH CARE PERSONNEL

**DOI:** 10.1093/geroni/igad104.2395

**Published:** 2023-12-21

**Authors:** Janne Bromseth

**Affiliations:** Høgskolen i Innlandet, Elverum, Ostfold, Norway

## Abstract

Person-centered care is the dominating care philosophical framework being used in geriatric care services in Norway today, as a point of departure for good practice. It implies that all care-receivers should be met from an empathic and individual perspective- and an increased focus on basic psychological needs. To be positively confirmed in relation to ones′ identities and to feel included in communities are central parts of this (Kitwood 1997). But is this also the case for older LGBTIQ+-adults? And which competence and knowledge on LGBTIQ+-adults′ life course experiences and aging conditions exist amongst health care staff working in long-term care facilities, home care services and day care activities? This paper is based on results from the interview study “Queer perspectives on geriatric care in Norway”, investigating experiences of person-centered care and inclusive practices from two perspectives; non-heterosexual care receivers and care providers in the Oslo-area. The analyzed material consists of: 1) interviews with 11 LGBQ-people between 61- 78 years old about their experiences with health care services over the life course 2) 3 focus groups and 2 individual interviews with health care staff working in a range of health and care services targeting 65+ adults. A majority of the interviewees had received training on LGBTIQ+-inclusive health care. The paper will mainly present results from the second study. What are leaders′ attitudes and knowledge regarding active work with gender and sexual diversity and equal access to care, and which experiences do they have from implementing LGBTIQ-awareness in policy and care practices?

